# Questionnaire results on exposure characteristics of pregnant women participating in the Japan Environment and Children Study (JECS)

**DOI:** 10.1186/s12199-018-0733-0

**Published:** 2018-09-15

**Authors:** Miyuki Iwai-Shimada, Shoji F. Nakayama, Tomohiko Isobe, Takehiro Michikawa, Shin Yamazaki, Hiroshi Nitta, Ayano Takeuchi, Yayoi Kobayashi, Kenji Tamura, Eiko Suda, Masaji Ono, Junzo Yonemoto, Toshihiro Kawamoto, the Japan Environment and Children’s Study Group, Toshihiro Kawamoto, Yukihiro Ohya, Reiko Kishi, Nobuo Yaegashi, Koichi Hashimoto, Chisato Mori, Shuichi Ito, Zentaro Yamagata, Hidekuni Inadera, Michihiro Kamijima, Takeo Nakayama, Hiroyasu Iso, Masayuki Shima, Yasuaki Hirooka, Narufumi Suganuma, Koichi Kusuhara, Takahiko Katoh

**Affiliations:** 1 Japan Environment and Children’s Study Programme Office, National Institute for Environmental Studies, 16-2 Onogawa, Tsukuba, 305-8506 Japan; 2 Department of Preventive Medicine and Public Health, Keio University, 35 Shinanomachi, Shinjuku-ku, Tokyo, 1608582 Japan; 3 Department of Environmental Health, University of Occupational and Environmental Health, 1-1 Iseigaoka, Yahatanishi-ku, Kitakyushu, 807-8555 Japan

**Keywords:** Birth cohort, Epidemiology, Exposure, Japan Environment and Children’s Study, JECS

## Abstract

**Background:**

The Japan Environment and Children’s Study (JECS) is a nation-wide birth cohort study investigating environmental effects on children’s health and development. In this study, the exposure characteristics of the JECS participating mothers were summarized using two questionnaires administered during pregnancy.

**Methods:**

Women were recruited during the early period of their pregnancy. We intended to administer the questionnaire during the first trimester (MT1) and the second/third trimester (MT2). The total number of registered pregnancies was 103,099.

**Results:**

The response rates of the MT1 and MT2 questionnaires were 96.8% and 95.1%, respectively. The mean gestational ages (SDs) at the time of the MT1 and MT2 questionnaire responses were 16.4 (8.0) and 27.9 (6.5) weeks, respectively. The frequency of participants who reported “lifting something weighing more than 20 kg” during pregnancy was 5.3% for MT1 and 3.9% for MT2. The Cohen kappa scores ranged from 0.07 to 0.54 (median 0.31) about the occupational chemical use between MT1 and MT2 questionnaires. Most of the participants (80%) lived in either wooden detached houses or steel-frame collective housing. More than half of the questionnaire respondents answered that they had “mold growing somewhere in the house”. Insect repellents and insecticides were used widely in households: about 60% used “moth repellent for clothes in the closet,” whereas 32% applied “spray insecticide indoors” or “mosquito coil or an electric mosquito repellent mat.”

**Conclusions:**

We summarized the exposure characteristics of the JECS participants using two maternal questionnaires during pregnancy.

**Electronic supplementary material:**

The online version of this article (10.1186/s12199-018-0733-0) contains supplementary material, which is available to authorized users.

## Background

The Japan Environment and Children’s Study (JECS) is a nation-wide birth cohort study initiated in 2011. JECS aims to investigate relationships between environmental factors and children’s health and development by recruiting 100,000 expectant mothers [1–3]. In JECS, children are followed from before birth to 13 years old. The exposures during the prenatal period were assessed using self-administered questionnaires and biological samples collected from the mothers during the first trimester, during the second/third trimester, and after delivery. Postnatal exposures were assessed mainly using questionnaires administered to the mothers every 6 months after birth [1].

Exposure assessment during the prenatal and postnatal period in a birth cohort study is critical to investigate the effect of the environment on children’s health because their developing organs are susceptible to various environmental factors [4]. Many birth cohort studies have been conducted aiming to illustrate the environmental effects on children’s health, including the Danish National Birth Cohort [5], the Norwegian Mother and Child Cohort Study (MoBa) [6, 7], Generation R in the Netherlands [8] and the Mothers’ and Children’s Environmental Health study in South Korea [9]. In JECS, the exposure assessment is based on four approaches: (1) questionnaires, (2) biomonitoring, (3) environmental measurements, and (4) simulation models [2, 3]. The current leading risk factors for the global disease burden are high blood pressure, tobacco smoking including second-hand smoke, household air pollution, and diet. Moreover, worldwide, the contribution of different risk factors to the disease burden has changed substantially, with a shift away from the risks of communicable diseases in children toward those of non-communicable diseases in adults [10]. At the same time, the causation of many chronic diseases and developmental disorders is poorly understood still. For example, the development and exacerbation of asthma can be associated with the complex interactions between environmental, social, and lifestyle factors (e.g., ambient air quality, house dust, mold, and smoking) as well as genetic and epigenetic factors [11]. Therefore, we should assess as many environmental exposures as possible in a birth cohort study instead of using a “one-exposure-one-health-effect” approach [12]. Not all exposures can be measured by biomonitoring or environmental monitoring. For some exposures, e.g., occupational history, daily consumer products, and dwelling condition, we had to rely on questionnaire for data collection. Since we had not found any standardized exposure questionnaire, we developed our own questionnaire for the use in JECS. Thus, it is important for us to characterize JECS exposure questionnaire data for the later use in the analysis of the association between environmental factors and children’s health. To our knowledge, this is the first to compare the responses of approximately 100,000 pregnant women to the exposure questionnaires administered twice during early and mid–late pregnancy periods. In this paper, we describe the environmental exposures of the JECS participants using two maternal questionnaires during pregnancy. We assessed whether pregnant women changed the environmental, lifestyle, and/or workload during pregnancy. The questionnaires were designed to collect information associated with chemical exposures such as dwelling conditions, indoor environment, usage of consumer products, and occupation.

## Methods

### Study protocol

**Fig. 1:**
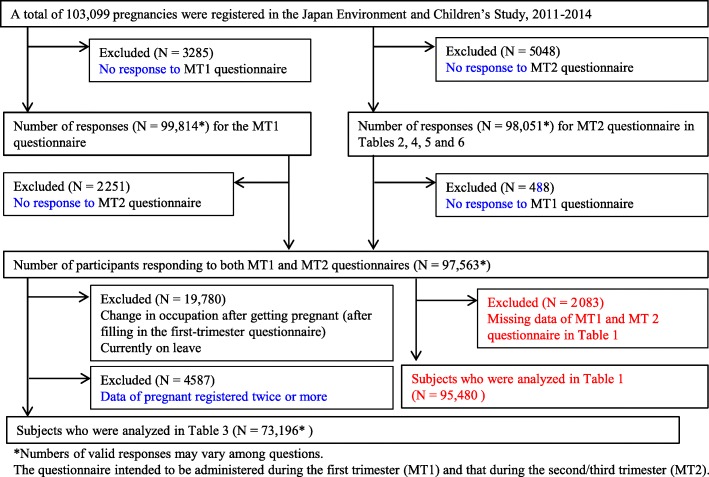
Environmental exposure data from questionnaires administered to first-trimester and second/third-trimester pregnant women in the Japan Environment and Children’s Study (JECS)

The JECS study protocol has been published elsewhere [1]. Briefly, 15 Regional Centers were selected to cover wide geographical areas in Japan, located from the north, Hokkaido, to the south, Okinawa [1]. The recruitment took place from January 2011 to March 2014. The eligibility criteria for participants (expecting mothers) were as follows: (1) They should reside in the study areas at the time of the recruitment and are expected to reside continually in Japan for the foreseeable future, (2) expected delivery date should be between 1 August 2011 and mid-2014, and (3) they should be capable to participate in the study without difficulty, i.e., must be able to comprehend the Japanese language and complete the self-administered questionnaire [1]. Self-administered questionnaires completed by the mothers during the first trimester and the second/third trimester were used to collect information on demographic factors, medical and obstetric history, physical and mental health, lifestyle, occupation, environmental exposure at home and in the workplace, housing conditions, and socioeconomic status. The baseline characteristics of the participants have been described elsewhere [2]. In this paper, we report the information about the use of chemical substances by mothers and their work/home environments using questionnaires administered during their pregnancy. We summarized two maternal questionnaires, i.e., the questionnaire intended to be administered during the first trimester (MT1) and that during the second/third trimester (MT2). The MT1 questionnaire collected information on activities and chemical use related to occupation during their pregnancy as exposure metrics. The MT2 questionnaire repeated the questions asked in the MT1 questionnaire and then collected data on their dwelling conditions, the indoor environment, and the use of consumer products (see Supplemental methods). The numbers of responses from the JECS participants for the MT1 and MT2 questionnaires are provided in Fig. 1. The total number of registered pregnancies was 103,099. The response rates of the MT1 and MT2 questionnaire were 96.8% and 95.1%, respectively. The mean gestational ages (SD) at the time of the MT1 and MT2 questionnaire responses were 16.4 (8.0) and 27.9 (6.5) weeks, respectively.

### Statistical analysis

The present study was based on the data set jecs-ag-20160424. Categorical variables were reported as a median with interquartile ranges, and categorical variables were the proportion of each questionnaire item to the total number of response. All analyses were performed using JMP version 12.2.0 (SAS Institute Inc., Cary, NC, USA), and *P* value < 0.0001 was considered statistically significant. We used the McNemar test to assess the differences in proportions between MT1 and MT2. The two questionnaires agreement was assessed using Cohen’s kappa coefficient (kappa scores) [13]. The  kappa score of 0–0.20 was characterized as poor agreement or no agreement beyond chance, 0.21–0.40 as fair, 0.41–0.60 as moderate, 0.61–0.80 as substantial, and 0.81–1.00 as almost perfect agreement [14].

## Results

The total number of pregnant women participating in JECS was 103,099. Michikawa et al. [2] have published previously the baseline characteristics of the JECS participants, including age at delivery, marital status, family composition, educational background, household income, and passive smoking (presence of smokers at home). The mean gestational ages (SD) at the time of the MT1 and MT2 questionnaire responses were 16.4 (8.0) and 27.9 (6.5) weeks, respectively.

Table 1 shows the workload characteristics during work and daily life at the current time and at any time since becoming pregnant. The numbers of participants who reported workloads of “lifting something weighing more than 20 kg” and “going in and out of commercial refrigerator or freezer” decreased significantly from the first trimester to the second/third trimester. In contrast, workloads of “exposed to loud noise” and “using manufacturing tools with vibration” increased significantly.

**Table 1:** Characteristics of workload from workplace, hobbies, and household during pregnancy as reported via two questionnaires of the MT1 and MT2 in the Japan Environment and Children’s Study (JECS)

Variables	MT1		MT2		*P*
	*N*	%	*N*	%	
I have been engaged in at least one of the following activities from nos. 1 to 7 after becoming pregnant					
Yes	13,410	14.0	11,306	11.8	< 0.0001
No	82,070	86.0	84,174	88.2	
1. Lifting objects that weigh more than 20 kg					
Yes	5078	5.3	3744	3.9	< 0.0001
No	90,402	94.7	91,736	96.1	
2. Exposed to loud noise					
Yes	3353	3.5	3597	3.8	< 0.0001
No	92,127	96.5	91,883	96.2	
3. Going in and out of commercial refrigerator or freezer					
Yes	2646	2.8	2091	2.2	< 0.0001
No	92,834	97.2	93,389	97.8	
4. Working in a hot place that makes one sweat					
Yes	1841	1.9	1719	1.8	0.0078
No	93,639	98.1	93,761	98.2	
5. Using organic solvent					
Yes	1508	1.6	1583	1.7	0.0288
No	93,972	98.4	93,897	98.3	
6. Handling powder dust					
Yes	810	0.8	850	0.9	0.1211
No	94,670	99.2	94,630	99.1	
7. Using manufacturing tools with vibration					
Yes	417	0.4	565	0.6	< 0.0001
No	95,063	99.6	94,915	99.4	

*P* values are by McNemar test. The questionnaire intended to be administered during the first trimester (MT1) and that during the second/third trimester (MT2)

*N* number of valid responses

Table 2 shows the frequencies of workload characteristics after becoming pregnant as reported in MT2. The frequency of “lifting something weighing more than 10 kg (including a child),” “using a tool/equipment or riding a vehicle with a strong vibration,” “going in and out of a commercial refrigerator or freezer,” and “working in a hot place that makes one sweaty” more than once a month were 67%, 1.6%, 4.5%, and 0.3%, respectively.

**Table 2:** Workload characteristics after becoming pregnant as reported via second/third trimester (MT2) questionnaire in the Japan Environment and Children’s Study (JECS)

Variables	*N*	%
Frequency of lifting something weighing more than 10 kg (including a child) after becoming pregnant	97,587	
Never	32,133	32.9
1–3 times a month	17,251	17.7
1–4 times a week	15,582	16.0
5 times a week or more	32,621	33.4
Living or working in a noisy environment after becoming pregnant	97,502	
No	87,260	89.5
Yes	10,242	10.5
Frequency of working sometime between 10 p.m. and dawn after becoming pregnant	97,491	
Never	89,394	91.7
1–3 times a month	4614	4.7
1–4 times a week	3002	3.1
5 times a week or more	481	0.5
Frequency of working in a hot place that makes one sweaty after becoming pregnant	97,472	
Never	89,385	91.7
1–3 times a month	3979	4.1
1–4 times a week	3059	3.1
5 times a week or more	1049	1.1
Frequency of going in and out of a commercial refrigerator or freezer after becoming pregnant	97,396	
Never	93,039	95.5
1–3 times a month	1506	1.6
1–4 times a week	1967	2.0
5 times a week or more	884	0.9
Frequency of using a tool/equipment or riding a vehicle with a strong vibration after becoming pregnant	97,453	
Never	95,911	98.4
1–3 times a month	939	1.0
1–4 times a week	383	0.4
5 times a week or more	220	0.2

*N* number of valid responses, *MT2* questionnaire administered to second/third-trimester pregnant women

Table 3 summarizes the occupational use of chemicals after becoming pregnant. Using a questionnaire similar to those used in MT1 and MT2 (for details see Additional file 1), Cohen’s kappa scores ranged from 0.07 to 0.54 (median 0.31). The kappa scores demonstrated mostly fair (between 0.21 and 0.4) to moderate (between 0.41 and 0.6) agreement between MT1 and MT2 except for the use of mercury and engine oil (poor, kappa scores up to 0.2).

**Table 3:** Frequency of the occupational use of chemicals for more than half a day during pregnancy (MT1 and MT2 questionnaires)

	MT1	MT2	*N*
	%	%	Kappa scores
Anti-cancer drug (not including your own remedy)			*N* = 63,576
No	98.7	98.8	0.54
1–3 times a month	0.8	0.9	
1–6 times a week	0.4	0.3	
Everyday	< 0.1	0.1	
Lead-free solder			*N* = 63,388
No	99.7	99.7	0.54
1–3 times a month	0.1	0.1	
1–6 times a week	0.1	0.2	
Everyday	0.1	0.1	
Any products containing lead like solder			*N* = 63,388
No	99.7	99.7	0.45
1–3 times a month	0.2	0.2	
1–6 times a week	0.1	0.1	
Everyday	0.1	0.1	
Formalin, formaldehyde			*N* = 63,584
No	99.2	99.2	0.44
1–3 times a month	0.5	0.5	
1–6 times a week	0.3	0.2	
Everyday	0.1	0.1	
Microbes			*N* = 63,399
No	99.6	99.6	0.44
1–3 times a month	0.2	0.2	
1–6 times a week	0.2	0.1	
Everyday	0.1	0.1	
General anesthetic for surgery at hospital			*N* = 63,611
No	99.2	99.1	0.42
1–3 times a month	0.4	0.5	
1–6 times a week	0.3	0.3	
Everyday	0.1	0.1	
Photo copying machine, laser printer			*N* = 64,895
No	70.6	66.1	0.39
1–3 times a month	8.1	11.4	
1–6 times a week	14.2	15.2	
Everyday	7.1	7.3	
Radiation, radioactive substances, isotopes			*N* = 63,385
No	98.1	98.5	0.38
1–3 times a month	0.9	0.7	
1–6 times a week	0.8	0.5	
Everyday	0.3	0.2	
Medical sterilizing disinfectant			*N* = 63,931
No	88.5	86.8	0.37
1–3 times a month	3.3	5.3	
1–6 times a week	6.0	5.8	
Everyday	2.3	2.0	
Dyestuffs (hair coloring)			*N* = 62,560
No	93.4	90.8	0.32
1–3 times a month	5.5	8.0	
1–6 times a week	0.6	0.7	
Everyday	0.4	0.5	
Permanent marker			*N* = 64,471
No	70.3	60.5	0.30
1–3 times a month	15.8	23.6	
1–6 times a week	11.1	13.2	
Everyday	2.8	2.7	
Paint			*N* = 63,569
No	80.0	72.9	0.29
1–3 times a month	10.2	15.5	
1–6 times a week	7.8	9.1	
Everyday	2.4	2.5	
Chromium, arsenic, cadmium			*N* = 63,386
No	99.9	99.9	0.28
1–3 times a month	< 0.1	< 0.1	
1–6 times a week	< 0.1	< 0.1	
Everyday	< 0.1	< 0.1	
Organic solvents			*N* = 63,471
No	92.9	91.1	0.27
1–3 times a month	5.4	7.2	
1–6 times a week	1.4	1.4	
Everyday	0.3	0.3	
Chlorine bleach, germicide			*N* = 64,016
No	81.1	73.7	0.27
1–3 times a month	13.2	19.7	
1–6 times a week	4.9	5.8	
Everyday	0.8	0.8	
Kerosene, petroleum, benzene, gasoline			*N* = 63,778
No	90.2	84.2	0.26
1–3 times a month	7.7	12.5	
1–6 times a week	2.0	3.2	
Everyday	0.1	0.1	
Insecticide			*N* = 63646
No	94.3	91.9	0.21
1–3 times a month	4.8	7.0	
1–6 times a week	0.9	1.0	
Everyday	0.1	0.1	
Herbicide			*N* = 62837
No	99.4	98.9	0.19
1–3 times a month	0.6	1.1	
1–6 times a week	< 0.1	< 0.1	
Everyday	< 0.1	< 0.1	
Engine oil			*N* = 63519
No	99.0	99.2	0.18
1–3 times a month	0.7	0.6	
1–6 times a week	0.2	0.2	
Everyday	0.1	0.1	
Mercury			*N* = 63,288
No	99.7	99.4	0.07
1–3 times a month	0.3	0.5	
1–6 times a week	< 0.1	< 0.1	
Everyday	< 0.1	< 0.1	
Agricultural chemical not listed above or unidentified			*N* = 64,388
No	99.8	No data	
1–3 times a month	0.1		
1– 6 times a week	< 0.1		
Everyday	< 0.1		
Other chemical substances			*N* = 64,313
No	99.1	No data	
1–3 times a month	0.2		
1–6 times a week	0.4		
Everyday	0.3		

The questionnaire intended to be administered during the first trimester (MT1) and that during the second/third trimester (MT2)

*N* number of valid responses

Table 4 presents the dietary habits during pregnancy as reported on the MT2 questionnaire. Frequency of eating “fast foods,” “retort pouch foods,” “instant noodles, soups, or other foods packed in plastic cups that can be cooked by pouring hot water,” and “canned foods” more than once a week were 15%, 23%, 21%, and 7%, respectively. Frequency of “eating pre-packed foods sold at convenience stores, supermarkets or box lunch shops,” “eating out at a restaurant or eating place,” and “eating frozen foods” more than once a week were 38%, 46%, and 33%, respectively.

**Table 4:** Dietary habits during pregnancy for breakfast, lunch, or dinner during the last month (MT2)

	*N*	%
Eating out at a restaurant or eating place	97,528	
Less than once a week	52,962	54.3
1–2 times a week	40,545	41.6
3–4 times a week	3261	3.3
5–6 times a week	601	0.6
Everyday	159	0.2
Eating pre-packed foods sold at convenience stores, supermarkets or box lunch shops	97,505	
Less than once a week	60,850	62.4
1–2 times a week	27,797	28.5
3–4 times a week	6485	6.7
5–6 times a week	1798	1.8
Everyday	575	0.6
Eating frozen foods	97,381	
Less than once a week	65,068	66.8
1–2 times a week	22,767	23.4
3–4 times a week	7313	7.5
5–6 times a week	1663	1.7
Everyday	570	0.6
Eating retort pouch foods	97,284	
Less than once a week	75,387	77.5
1–2 times a week	20,012	20.6
3–4 times a week	1668	1.7
5–6 times a week	170	0.2
Everyday	47	< 0.1
Eating instant noodles, soups, or other foods packed in plastic cups that can be cooked by pouring hot water	97,277	
Less than once a week	77,380	79.5
1–2 times a week	17,758	18.3
3–4 times a week	1869	1.9
5–6 times a week	213	0.2
Everyday	57	0.1
Fast-food intake (e.g., French fries, pizza, donuts)	97,367	
Less than once a week	82,699	84.9
1–2 times a week	13,845	14.2
3–4 times a week	736	0.8
5–6 times a week	71	0.1
Everyday	16	< 0.1
Eating canned foods	96,915	
Less than once a week	89,919	92.8
1–2 times a week	6662	6.9
3–4 times a week	288	0.3
5–6 times a week	32	< 0.1
Everyday	14	< 0.1

*N* Number of valid responses

Table 5 presents the household environment characteristics such as dwelling condition, air conditioning, cleanup, and mobile phone use during pregnancy collected via the MT2 questionnaire. Most of the participants (80%) lived in either wooden detached houses or steel-frame collective housing. The proportion of the respondents living in a housing that was over 20 years old was 35%. More than half of the questionnaire respondents answered that they had “mold growing somewhere in the house,” with the bathroom being the most frequent site of mold. Wooden floors (covered by carpets, tiles, or no covering) were present in 78% of the residences. As for household cleaning, 92% of the participants had been vacuuming more than once a week. The proportion of participants who did not have a mobile phone was 0.1–0.2%.

**Table 5:** Household environment characteristics during pregnancy (MT2)

Category	Variables	*N*	Median	%
			(25th–75th percentiles)	
Dwelling condition and material	Type of residence	97,315		
	Wooden detached house	40,269		41.4
	Steel-frame detached house	6190		6.4
	Wooden multiple-dwelling house/apartment	12,042		12.4
	Steel-frame multiple-dwelling house/apartment	37,861		38.9
	Others	953		1.0
	Age of house/apartment building	97,238		
	< 1 year	5432		5.6
	1 ≦ year < 3	10,920		11.2
	3 ≦ year < 5	9152		9.4
	5 ≦ year < 10	14,903		15.3
	10 ≦ year < 20	22,610		23.3
	20 years ≦	24,576		25.3
	Unknown	9672		9.9
	Number of years living in the current place of residence (years)	94,899	3 (1–5)	
	Floor living on/number of floors in the apartment building	63,509/67,230	2 (1–3)/2 (2–4)	
	Number of rooms in the house/apartment	97,293	3 (3–5)	
	Size of the floor space of the house/apartment (m^2^)	40,321	67 (50–100)	
	House renovation/interior finishing after getting pregnant	97,242		
	Yes (%)	3076		3.2
	Living in an all-electric house/building	97,276		
	Yes (%)	18,317		18.8
	Small refuse incinerator on the premises of home	97,408		
	Yes, but it is no longer used (%)	1298		1.3
	Yes, it is used still (%)	2632		2.7
	Use of a water purifier on a water faucet	97,427		
	Yes (%)	27,539		28.3
Mold	Mold growing somewhere in the house	96,853		
	Yes (%)	60,946		62.9
	Number of responses	98,051		
	Kitchen (yes, %)	10,869		11.1
	Living room (yes, %)	2020		2.1
	Mother’s bedroom (yes, %)	5306		5.4
	Other bedroom (yes, %)	1122		1.1
	Bathroom (yes, %)	57,252		58.4
	Lavatory (yes, %)	4278		4.4
	Other place (yes, %)	2886		2.9
Pet	Having a pet currently	97,538		
	Yes (%)	22,483		23.1
	Number of responses	98,051		
	Cat (yes, %)	6852		7.0
	Bird (yes, %)	682		0.7
	Dog (kept in- and outside of residence, yes, %)	13,597		13.9
	Hamster (yes, %)	1018		1.0
	Turtle (yes, %)	1166		1.2
	Others (yes, %)	4076		4.2
Air conditioning	Appliance mainly used to cool rooms in the house/apartment	97,618		
	Air conditioner	70,702		72.4
	Electric fan	24,223		24.8
	Others	281		0.3
	Nothing	2412		2.5
	Use of a humidifier during the last year	97,634		
	Yes (%)	56,469		57.8
	Use of a dehumidifier/dehumidifying function of an air conditioner during the last year	97,564		
	Yes (%)	58,808		60.3
	Use of an air-cleaning device	97,632		
	Yes (%)	50,235		51.5
	Heating appliance used in the living room during winter (yes, %)	92,257		
	Yes (%)	91,587		99.3
	Type of heating equipment in living room	98,051		
	Kerosene heater/kerosene fan heater	48,454		49.4
	Gas heater/gas fan heater	7800		8.0
	Kerosene/gas heater (with a chimney or an exhaust pipe that reaches outside of house)	1514		1.5
	Air conditioner/steam heater/oil heater	53,741		54.8
	Electric “kotatsu” (a table with an electric heater underneath, with a quilt)/electric heater/electric carpet/other electric heating equipment	58,347		59.5
	Central heating/floor heating	5831		5.9
	Charcoal/briquette “kotatsu” or “hibachi” (Japanese heating appliance using charcoal as fuel)	669		0.7
	Other equipment	2404		2.5
	Use of any equipment to heat a bed during winter	96,376		
	Yes (%)	30,262		31.4
	Type of heating equipment in bed	98,051		
	Electric “anka” (bed warmer)	2969		3.0
	Electric blanket	12,608		12.9
	Hot water bottle	16,351		16.7
	Other equipment	1800		1.8
Cleaning	Materials covering the flooring of the living room	97,475		
	Tatami (Japanese straw floor covering)	11,285		11.6
	Carpet on tatami	8853		9.1
	Flooring/wooden flooring/tiles	34,574		35.5
	Carpet on flooring/wooden flooring/tiles	40,990		42.1
	Other	1773		1.8
	Frequency of cleaning the floor of the living room with a vacuum cleaner^a^	97,616		
	Everyday	17,156		17.6
	A few times a week	42,918		44.0
	Once a week	29,605		30.3
	1–2 times a month	5784		5.9
	A few times a year	915		0.9
	Almost never or never	1238		1.3
	Frequency of cleaning the floor of the bedroom with a vacuum cleaner^a^	97,617		
	Everyday	10,824		11.1
	A few times a week	38,693		39.6
	Once a week	34,392		35.2
	1–2 times a month	10,371		10.6
	A few times a year	1718		1.8
	Almost never or never	1619		1.7
	Frequency of cleaning the “futon” (Japanese mattress and blanket for bedding) with a vacuum cleaner^a^	97,451		
	A few times a week	3797		3.9
	Once a week	10,763		11.0
	1–2 times a month	16,369		16.8
	A few times a year	12,190		12.5
	Almost never or never	54,332		55.8
	Frequency of airing the “futon” (Japanese mattress and blanket for bedding)^a^	97,446		
	A few times a week	8595		8.8
	Once a week	23,081		23.7
	1–2 times a month	36,214		37.2
	A few times a year	18,216		18.7
	Almost never or never	11,340		11.6
	Use of anti-mite covers for “futon” or bedding after getting pregnant	96,946		
	Yes (%)	7767		8.0
Outdoor time	Spending time outdoors (hours per day)	93,944	1.0 (1.0–2.0)	
Mobile phone	Talk time (per day)	97,648		
	I do not have a mobile phone	144		0.1
	None	10,011		10.3
	Less than 10 min	69,381		71.1
	For 10–60 min	15,722		16.1
	More than 1 h	2390		2.4
	Number of emails sent and received (per day)	97,606		
	I do not have a mobile phone	154		0.2
	None	2009		2.1
	Less than 10 times	83,153		85.2
	More than 10 times	12,290		12.6

*N* number of responses

^a^Average throughout the year

Table 6 shows the use of household chemicals during pregnancy (MT2). Most of the participants used a deodorizer or an air freshener, especially in the lavatory. Insect repellents and insecticides were used widely in households: about 60% used “moth repellent for clothes in the closet,” whereas 32% applied “spray insecticide indoors” or “mosquito coil or an electric mosquito repellent mat.” About 40% of the participants had used “medicated soap or antibacterial soap,” “cosmetics with strong perfume or a fragrance,” and “nail polish” at least once since becoming pregnant. The incidence of “coloring or perming hair at a beauty salon” during pregnancy was 50%. Combined with the frequency of “coloring or perming hair at home,” the results indicate that most subjects carried out hair treatments during pregnancy.

**Table 6:** The use of household chemicals during pregnancy (MT2)

Variables	*N*	%
Frequency of refueling a car with gasoline at a self-service gas station	97,672	
Everyday	147	0.2
4–6 times a week	258	0.3
2–3 times a week	2354	2.4
Once a week	8957	9.2
1–3 times a month	31,912	32.7
Less than once a month	19,518	20.0
Never	34,526	35.3
Use of a deodorizer or an air freshener		
Lavatory	97,531	
Yes (%)	82,658	84.8
Living room or bedroom	97,495	
Yes (%)	55,267	56.7
Use of a moth repellent for clothes in the closet	97,513	
Yes, continuously	21,041	21.6
Yes, sometimes	36,626	37.6
Never	39,846	40.9
Use of a spray insecticide indoors	96,799	
Yes (%)	30,843	31.9
Frequency of using a spray insecticide indoors	31,676	
Everyday	572	1.8
A few times a week	3490	11.0
Once a week	1962	6.2
1–3 times a month	6368	20.1
Less than once a month	19,284	60.9
Use of a mosquito coil or an electric mosquito repellent mat^a^	97,187	
Yes (%)	30,897	31.8
Frequency of using a mosquito coil or electric mosquito repellent mat^a^	31,282	
Everyday	8986	28.7
A few times a week	10,943	35.0
Once a week	2175	7.0
1–3 times a month	4193	13.4
Less than once a month	4985	15.9
Use of a liquid insecticide for maggot and mosquito larva	97,618	
Yes (%)	710	0.7
Frequency of using a liquid insecticide for maggot and mosquito larva	706	
Everyday	27	3.8
A few times a week	66	9.3
Once a week	56	7.9
1–3 times a month	139	19.7
Less than once a month	418	59.2
Use of an herbicide or a gardening pesticide in a garden, balcony, or farm	97,425	
Yes (%)	8600	8.8
Frequency of using an herbicide or a gardening pesticide in a garden, balcony, or farm	8534	
Everyday	83	1.0
A few times a week	201	2.4
Once a week	211	2.5
1–3 times a month	1363	16.0
Less than once a month	6676	78.2
Spraying insect repellent on clothes or putting lotion on skin	97,152	
Yes (%)	23,829	24.5
Frequency of spraying insect repellent on clothes or putting lotion on skin	24,127	
Everyday	517	2.1
A few times a week	4701	19.5
Once a week	2134	8.8
1–3 times a month	5592	23.2
Less than once a month	11,183	46.4
Use of smoke insecticide indoors	97,500	
Yes (%)	6578	6.7
Use of a waterproof spray on clothes or shoes	97,468	
Yes (%)	11,005	11.3
Use of medicated soap or antibacterial soap	97,339	
Yes (%)	41,178	42.3
Use of a body deodorant	97,430	
Yes (%)	32,951	33.8
Use of cosmetics with strong perfume or a fragrance	97,588	
Quite often	2737	2.8
Sometimes	14,613	15.0
Rarely	19,465	19.9
Never	60,773	62.3
Manicuring or using nail polish	97,608	
Quite often	5647	5.8
Sometimes	18,313	18.8
Rarely	14,332	14.7
Never	59,316	60.8
Use of hair coloring products (e.g., hair dye) or perm solutions at home	97,616	
Quite often	1246	1.3
Sometimes	11,801	12.1
Rarely	9185	9.4
Never	75,384	77.2
Coloring or perming hair at a beauty salon	97,585	
Quite often	3167	3.2
Sometimes	28,750	29.5
Rarely	17,100	17.5
Never	48,568	49.8
Use of sunscreen	97,635	
Quite often	31,144	31.9
Sometimes	27,038	27.7
Rarely	9622	9.9
Never	29,831	30.6
Using drug for treatment of scabies or lice	97,613	
Yes (%)	558	0.6

*N* number of valid responses

^a^Continuously for more than a few hours

## Discussion

We developed an in-house exposure questionnaire for the use in JECS since there were no standardized ones available. Almost two identical questionnaires were administered during pregnancy. The exposure data included dwelling conditions, indoor environment, daily life consumer product uses, and occupation. To our knowledge, this is the first of its kind in Japan to characterize over 100,000 pregnant women’s exposure data by the questionnaire. The mean gestational age (SD) at the time of the MT1 questionnaire responses was 16.4 (8.0), which means about half of the participants responded the MT1 questionnaire during the second-trimester period of pregnancy or later. We intended to recruit the participants in early pregnancy but did not restrict to be in the first trimester. Some of the participants were registered at their mid to late pregnancy. When we exclude the responses from the mothers who responded during their gestational ages greater than 16 weeks from the MT1 questionnaire data analysis, the results were similar to those presented in Table 1 (data not shown). The timing of the questionnaire response must be taken into account when researchers use the MT1 questionnaire data for later analysis.

Most of the participants had little occupational exposure to chemicals during pregnancy, while 30–40% of the participants reported the use of personal care products and household pesticide application. Of the participants, 20–30% had consumed convenience foods such as fast foods and retort pouch foods more than once a week within the month prior to the survey, suggesting exposure to chemicals in preservatives or food-packaging materials such as phthalates and bisphenols. Phthalates and bisphenols are suspected endocrine disrupters and have been adversely associated with child health. This information can be used not only to analyze the association between environmental factors and children’s health but also in the future planning of the JECS exposure assessment using biomonitoring.

The Danish National Birth Cohort reported that heavy object lifting was associated with an increased risk of preterm birth in a dose–response manner [15]. Although no exposure–response relationship was observed for fetal death, Mocevic et al. [16] found an increased risk of stillbirth (fetal death ≥ 22 gestational weeks) among those who lifted more than 200 kg/day. In the Danish National Birth Cohort, 16,604 women (26.4%) carried heavy loads (> 20 kg) at work and 475 women (2.9%) lifted more than 1000 kg per day [15]. The Labor Standards Act protects pregnant Japanese women aged ≥ 18 years from tasks that involve heavy object lifting (continuing work, > 20 kg; intermittent work, > 30 kg). In JECS, only 5078 (5.3%) women in the MT1 questionnaire and 3744 (3.9%) women in the MT2 questionnaire lifted loads greater than 20 kg at work (Table 1), though most women in JECS lifted loads greater than 10 kg (including a child) (Table 2).

Various case-control studies have shown the relationship between maternal occupational exposure to solvents and some subtypes of malformations, mostly oral clefts [17–20]. Significant associations were also reported between maternal exposure to solvents and cardiac malformations [21, 22] and neural tube defects [20]. A review of the results of 49 studies showed that maternal occupational exposure to chemicals (lead and pesticides) was associated with time to pregnancy [23]. Snijder et al. [24] observed in the Netherlands (the Generation R Study) that maternal occupational exposure to polycyclic aromatic hydrocarbons, phthalates, alkylphenolic compounds, and pesticides influenced adversely several domains of fetal growth (fetal weight). In JECS, the occupational use of insecticides, organic solvents, and metals (sum of chromium, arsenic and cadmium, lead, and mercury) more than once a month was reported by 7.1%, 5.8%, and 0.6% of the participants, respectively (Table 3). These frequencies were slightly higher than those in the Generation R Study (*n* = 4680) in which the prevalence of maternal occupational use of pesticides, organic solvents, and metals were 0.5%, 4.7%, and 1.1%, respectively [24]. With the exception of mercury, occupational exposure to these chemicals was more prevalent in the JECS participants than in the Generation R participants.

Though exposure information obtained from questionnaires could be considered also an important variable, there are few validated standard questionnaire sets. As shown in Table 3, the kappa-coefficients demonstrate mostly fair to moderate agreement between the MT1 and MT2 questionnaires. Since all kappa scores resulted in < 0.61, it suggested that pregnant women could change the chemical use under occupation during pregnancy.

The National Health and Nutrition Survey of Japan [25] reported that the frequency of eating out at a restaurant was 25.1% in total women, 47.3% in women 20–29 years old, and 40.4% in women 30–39 years old. The survey reported also that the frequency of eating pre-packed foods was 39.4% in total women more than 20 years old. In JECS, the frequencies of eating out and eating pre-packed foods more than once a week were 45.7% and 37.6%, respectively. This result is similar to that of the National Health and Nutrition Survey in Japan, indicating that this part of the questionnaire is valid also.

The 2013 Housing and Land Survey of Japan reported that the proportions of wooden housing and non-wooden, such as steel-frame, housing were 58% and 42%, respectively [26]. The JECS results were similar to those of that survey with wooden and non-wooden dwellings reported by 54% and 45% of participants, respectively. In 1981, the Building Standards Act of Japan was revised to enforce new earthquake-resistance standards. The proportion of housing built after 1981 was 64.9% in the national survey (2013), while that of housing less than 20 years of age was 64.8% in JECS. The mean number of rooms and dwelling area in the national survey (2013) were 4.59 rooms and 94.42 m^2^ per house, respectively. The mean number of rooms and dwelling area in JECS were 3.89 rooms and 82.32 m^2^ per house, respectively. These results showed that the JECS participants lived in smaller and relatively newer houses compared with respondents to the national survey (2013).

In the questionnaire-based maternal environmental exposure assessment (*n* = 987) of the INTERGROWTH-21st Project, the rate of household pesticide application was 7.1% (70/987) in respondents from Brazil, China, India, Italy, Kenya, Oman, UK, and the USA [27]. In JECS, the rates of maternal use of moth repellent for clothes, indoor insecticide spray, mosquito coils/mats, liquid insecticides, smoke insecticides, and herbicides were 59%, 32%, 32%, 0.7%, 6.7%, and 8.8%, respectively. People in Japan appear to use more types of pesticides and to use them at a higher rate than people in the abovementioned countries. This indicates the importance of biomonitoring of pesticide chemicals in JECS.

There are some limitations of the JECS exposure assessment questionnaires. Firstly, the self-administered questionnaires were developed in-house by the JECS Programme Office and did not go through any validation process using biological or environmental measurements. Much of the exposure data could only be obtained using questionnaires; the accuracy and reliability of which could not be evaluated. However, some of our results were similar to those of national surveys on such topics as dwelling conditions and dietary habits; accordingly, we assumed that these parts of our questionnaires, at least, were somewhat reliable. The other topics had not been studied previously in Japan in either national surveys or scientific publications. To our knowledge, therefore, these results constitute the first report on the exposure status of pregnant women in Japan. Secondly, we investigated the two questionnaires reliability by administering nearly identical questionnaires in MT1 and MT2. However, there were subtle differences in how the questions were expressed in the MT1 and MT2 questionnaires (for details see Additional file 1), which may have affected the responses. In a future study, we plan to verify the questionnaire as thoroughly as possible using quantitative instruments such as biomonitoring and environmental measurements. Lastly, there were some extreme values observed among the questionnaire responses, e.g., 99 years for the “number of years living in the current place of residence,” 91/83 as “the floor living on/number of floors in the apartment building,” 93 for the “number of rooms in the house/apartment,” and 999 m^2^ for the “size of the floor space of the house/apartment.” Such values were observed in less than 0.01% of cases. We did not exclude these possible outliers from the analysis presented in this paper since there was no way for us to verify the accuracy of these responses.

This result will be used to design future JECS exposure assessments with biomonitoring. The questionnaire data will also be used to investigate the associations between environmental factors and children’s health and development when data comes available. Some parts of the questionnaire will be validated using biomonitoring data. Such questionnaire items are of great importance for other epidemiological and exposure studies since there are few validated exposure questionnaires. The validate questionnaire can also be used for a national biomonitoring program as a tool to collect exposure source information.

## Conclusions

We characterized the environmental exposures of the JECS participants using two maternal questionnaires. Most of the mothers had little occupational exposure to chemicals during pregnancy. The household use of pesticides was more frequent in JECS than in studies in other countries. It will also be used to investigate the associations between environmental factors and children’s health in the future.

## Additional file

Additional file 1: Supplementary information about questionnaire items for Tables 1 to 6. (PDF 126 kb)

## Abbreviations

JECS: Japan Environment and Children’s Study
MT1: First trimester
MT2: Second/third trimester
