# Supplementary material for: Questionnaire results on exposure characteristics of pregnant women participating in the Japan Environment and Children Study (JECS)
Source: Environ Health Prev Med. 2018 Sep 15;23:45. doi: 10.1186/s12199-018-0733-0 (PMC6138908; doi:10.1186/s12199-018-0733-0)
Supplement: Supplementary file 1 — Supplementary information about questionnaire items for Tables 1 to 6. (PDF 126 kb) [file 12199_2018_733_MOESM1_ESM.pdf]

Supplementary Methods for

Questionnaire results on exposure characteristics of pregnant women participating in the Japan Environment and Children Study (JECS)

Miyuki Iwai-Shimada, Shoji F. Nakayama*, Tomohiko Isobe, Takehiro Michikawa, Shin Yamazaki, Hiroshi Nitta, Ayano Takeuchi, Yayoi Kobayashi, Kenji Tamura, Eiko Suda, Masaji Ono, Junzo Yonemoto, Toshihiro Kawamoto, and the Japan Environment and Children's Study Group

*correspondence to: fabre@nies.go.jp

This PDF file includes:

Methods

Supplemental information of question list for Table 1 to Table 6

Methods

Supplemental information of question list for Table 1 to Table 6 were shown.

Supplemental information of question list for Table 1 to Table 6

1. Question list for Table 1 (same questions were used in MT1 and 2)

1-1. I have been engaged in at least one of the following activities (a to g) during work and daily life at the current time and at any time since becoming pregnant.

□Yes □No

↓

Check all that apply

□ a. Lifting something weighing more than 20kg

□ b. Using organic solvent (e.g., paint thinner, glue, etc.)

□ c. Being exposed to loud noise

□ d. Using manufacturing tools with vibration

□ e. Going in and out of commercial refrigerator or freezer

□ f. Working in a hot place making one sweaty

□ g. Working in a dusty environment

2. Question list for Table 2 (only MT2 questionnaire)

2-1. Frequency of lifting something weighing more than 10kg (including a child) after becoming pregnant (check one only)

□ Never

□ 1–3 times a month

□ 1–4 times a week

□ 5 times a week or more

2-2. Living or working in a noisy environment after becoming pregnant

□Yes □No

2-3. Frequency of going in and out of a commercial refrigerator or freezer after becoming pregnant (check one only)

□ Never

□ 1–3 times a month

□ 1–4 times a week

□ 5 times a week or more

2-4. Frequency of working in a hot place that makes one sweaty after becoming pregnant (check one only)

□ Never

□ 1–3 times a month

□ 1–4 times a week

□ 5 times a week or more

2-5. Frequency of using a tool/equipment or riding a vehicle with a strong vibration after becoming pregnant (check one only)

□ Never

□ 1–3 times a month

□ 1–4 times a week

□ 5 times a week or more

2-6. Frequency of working sometime between 10 pm and dawn after becoming pregnant (check one only)

□ Never

□ 1–3 times a month

□ 1–4 times a week

□ 5 times a week or more

3. Question list for Table 3 (similar questions were used in MT1 and 2)

MT1 question: frequency of using or handling the following materials (a to v) during work for more than half a day since becoming pregnant

MT2 question: frequency of using or handling the following materials (a to v) during work for more than half a day from becoming pregnant to the present

	Never	1 to 3 times a month	1 to 6 times a week	Everyday
ex. Kerosene, Petroleum, Benzene, Gasoline	○1	2	3	4
a. Kerosene, Petroleum, Benzene, Gasoline	1	2	3	4
b. Chlorine bleach, Germicide (with the warning, “Do not mix. Hazardous.”)	1	2	3	4
c. Medical disinfectant	1	2	3	4
d. Permanent marker	1	2	3	4

e. Water-based paint or Inkjet printer	1	2	3	4
f. Organic solvents (e.g., paint thinner, solvent for examination/analysis/extraction, dry-cleaning detergent, stain-removing agent, paint coating, nail polish remover, etc.)	1	2	3	4
g. Photocopier, Laser printer	1	2	3	4
h. Engine oil	1	2	3	4
i. Formalin, Formaldehyde	1	2	3	4
j. Anticancer drug (excluding those prescribed to the respondent)	1	2	3	4
k. General anesthetic for surgery	1	2	3	4
l. Insecticide	1	2	3	4
m. Herbicide	1	2	3	4
n. Unidentified or other agricultural chemical	1	2	3	4
o. Radiation, Radioactive substances, Isotopes	1	2	3	4
p. Microbes	1	2	3	4
q. Any products containing lead (e.g., solder)	1	2	3	4
r. Lead-free solder	1	2	3	4
s. Chromium, Arsenic, Cadmium	1	2	3	4
t. Mercury	1	2	3	4
u. Dyestuffs (for hair coloring)	1	2	3	4
v. Other chemical substances	1	2	3	4

4. Question list for Table 4

4-1. Frequency of eating “fast food” (e.g., French fries, pizza, donuts) for breakfast, lunch, or dinner during the last month (check one only).

□Less than once a week

□1-2 times a week

□3-4 times a week

□5-6 times a week

□Everyday

4-2. Frequency of eating out for breakfast, lunch, or dinner at a restaurant or eating place during the past month (check one only).

□Less than once a week

□1-2 times a week

□3-4 times a week

□5-6 times a week

□Everyday

4-3. Frequency of eating pre-packed convenience foods sold at convenience stores, supermarkets or box lunch shops for breakfast, lunch or dinner during the past month (check one only).

□Less than once a week

□1-2 times a week

□3-4 times a week

□5-6 times a week

□Everyday

4-4. Frequency of eating frozen foods for breakfast, lunch or dinner during the past month (check one only).

□Less than once a week

□1-2 times a week

□3-4 times a week

□5-6 times a week

□Everyday

4-5. Frequency of eating retort pouch foods for breakfast, lunch or dinner during the past month (check one only).

□Less than once a week

□1-2 times a week

□3-4 times a week

□5-6 times a week

□Everyday

4-6. Frequency of eating instant noodles, soups, or other foods packed in plastic cups that can be cooked by pouring hot water for breakfast, lunch or dinner during the past month (check

one only).
□Less than once a week
□1-2 times a week
□3-4 times a week
□5-6 times a week
□Everyday

4-7. Frequency of eating canned foods for breakfast, lunch or dinner during the past month (check one only).
□Less than once a week
□1-2 times a week
□3-4 times a week
□5-6 times a week
□Everyday

5. Question list for Table 5

Please tell us about your home, home environment and dwelling conditions.

5-1. Type of residence (check one only)
□Wooden detached house
□Steel-frame detached house
□Wooden multiple-dwelling house/apartment
□Steel-frame multiple-dwelling house/apartment
□Other

5-2. Age of house/apartment building (check one only)
□Less than 1 year
□1 year or more and less than 3 years
□3 years or more and less than 5 years
□5 years or more and less than 10 years
□10 years or more and less than 20 years
□20 years or more
□Unknown

5-3. How long have you lived in your current place of residence？

Number of years living in the current place of residence

_________years

5-4. How many floors does your apartment building have? And what floor do you live on?
Number of floors in the apartment building
Floor living on

Living on the ________ floor of _______stories

5-6. How many rooms does your house/apartment have, excluding kitchen, bathroom, washroom, lavatory, warehouse and shuttle door?
Number of rooms in the house/apartment

_________rooms

5-7. What size is the floor space in your house/apartment?
Size of the floor space of the house/apartment

_________m^2

5-8. House renovation/interior finishing after getting pregnant

□Yes □No

5-9. Appliance mainly used to cool rooms in the house/apartment (check one only).

□Air conditioner □Electric fan □Other □No appliance was used

5-10. Use of a humidifier during the last year

□Yes □No

5-11. Use of a dehumidifier/dehumidifying function of an air conditioner during the last year

□Yes □No

5-12. Use of an air-cleaning device

□Yes □No

5-13. Heating appliance used in the living room during winter

□ Use a heating appliance □Use no heating appliance

↓

Check all that apply

□ Kerosene heater/Kerosene fan heater

□ Gas heater/Gas fan heater

□ Kerosene/Gas heater (with a chimney or an exhaust pipe that reaches outside of house)

□ Air conditioner/Steam heater/Oil heater

□ Electric “kotatsu” (A table with an electric heater underneath, with a quilt)/Electric heater/Electric carpet/other electric heating equipment

□ Central heating/Floor heating

□Charcoal/Briquette “kotatsu” or “hibachi” (Japanese heating appliance using charcoal as fuel)

□ Other heating appliance

5-14. Use of any equipment to heat a bed during winter

□Yes □No

↓

Check all that apply

□ Electric “anka” (bed warmer)

□ Electric blanket

□ Hot water bottle

□ Other equipment

5-15. Materials covering the flooring of the living room (check one only)

□ Tatami (Japanese straw floor covering)

□ Carpet on tatami

□ Flooring/Wooden flooring/Tiles

□ Carpet on flooring/wooden flooring/tiles

□ Other

5-16. Frequency of cleaning the floor of the living room with a vacuum cleaner (average throughout the year) (check one only)

□ Everyday

□ A few times a week

□ Once a week

□ 1-2 times a month

□ A few times a year

□ Almost never or never

5-17. Frequency of cleaning the floor of the bedroom with a vacuum cleaner (average

throughout the year) (check one only)

□ A few times a week

□ Once a week

□ 1-2 times a month

□ A few times a year

□ Almost never or never

5-18. Frequency of airing the "futon" (Japanese mattress and blanket for bedding) (average throughout the year) (check one only)

□ A few times a week

□ Once a week

□ 1-2 times a month

□ A few times a year

□ Almost never or never

5-19. Use of anti-mite covers for "futon" or bedding after getting pregnant

□Yes □No

5-20. Mold growing somewhere in the house

□Yes □No

↓

Check all that apply

□ Kitchen

□ Living room

□ Mother's bedroom

□ Other bedroom

□ Bathroom

□ Lavatory

□ Other place

5-21. Having a pet currently

□Yes □No

↓

Check all that apply

□ Cat

□ Bird

□ Dog (kept inside of residence)

□ Dog (kept outside of residence)

□ Hamster

□ Tortoise

□ Other pet

5-22. Living in an all-electric house/building

□Yes □No

5-23. A small refuse incinerator on the premises of home (check one only)

□No, □Yes, but it is no longer used □Yes, it is used currently.

5-24. Use of a water purifier on a water faucet (check one only)

□Yes □No □Other

5-25. How many hours do you spend outdoors on an average day?

Average number of hours spent outdoors

_________hours per day

5-26. Average length of talking on a mobile phone per day (check one only)

□ I don't have a mobile phone

□ 0 minutes

□ Less than 5 minutes

□ 5-9 minutes

□ 10-29 minutes

□ 30-59 minutes

□ 1 hour or more

5-27. Average number of emails/short messages sent/received on a mobile phone per day (check one only)

□ I don't have a mobile phone

□ 0 times

□ 1-2 times

□ 3-5 times

□ 6-9 times

□ More than 10 times

6. Question list for Table 6

6-1. Frequency of refueling a car with gasoline at a self-service gas station after getting pregnant (check one only)

□Everyday

□4-6 times a week

□2-3 times a week

□Once a week

□1-3 times a month

□Less than once a month

□Never

6-2. Use of a deodorant or an air freshener in the lavatory after getting pregnant

□Yes □No

6-3. Use of a deodorant or an air freshener in the living room or bedroom after getting pregnant

□Yes □No

6-4. Use of a moth repellent for clothes in the closet after getting pregnant (check one only)

□Yes, I have used one continuously □Yes, I sometimes use one □No

6-5. Use of a spray insecticide indoors after getting pregnant

□Yes □No

↓check one only

Frequency of using a spray insecticide indoors after getting pregnant

(check one only)

□Everyday

□A few times a week

□Once a week

□1-3 times a month

□Less than once a month

6-6. Use of a mosquito coil or an electric mosquito repellant mat continuously for more than a

few hours after getting pregnant

□Yes □No

↓check one only

Frequency of using a mosquito coil or electric mosquito repellant mat continuously for more than a few hours after getting pregnant (check one only)

□ Everyday

□ A few times a week

□ Once a week

□ 1-3 times a month

□ Less than once a month

6-7. Use of a liquid insecticide for maggot and mosquito larva after getting pregnant

□Yes □No

↓check one only

Frequency of using a liquid insecticide for maggot and mosquito larva after getting pregnant (check one only)

□ Everyday

□ A few times a week

□ Once a week

□ 1-3 times a month

□ Less than once a month

6-8. Use of an herbicide or a gardening pesticide in a garden, balcony, or farm after getting pregnant

□Yes □No

↓check one only

Frequency of using an herbicide or a gardening pesticide in a garden, balcony, or farm after getting pregnant (check one only)

□ Everyday

□ A few times a week

□ Once a week

□ 1-3 times a month

□ Less than once a month

6-9. Spraying insect repellent on clothes or putting lotion on skin after getting pregnant

□Yes □No

↓check one only

Frequency of spraying insect repellent on clothes or putting lotion on skin after getting pregnant (check one only)

□ Everyday

□ A few times a week

□ Once a week

□ 1-3 times a month

□ Less than once a month

6-10. Use of a smoke insecticide indoors after getting pregnant

□Yes □No

6-11. Use of a waterproof spray on clothes or shoes after getting pregnant

□Yes □No

6-12. Use of medicated soap or antibacterial soap after getting pregnant

□Yes □No

6-13. Use of a body deodorant after getting pregnant

□Yes □No

6-14. Use of cosmetics with strong perfume or a fragrance after getting pregnant （check one only）

□Quite often □Sometimes □Rarely □Never

6-15. Manicuring or using nail polish after getting pregnant (check one only)

□Quite often □Sometimes □Rarely □Never

6-16. Use of hair coloring products (e.g., hair dye) or perm solutions at home after getting pregnant (check one only)

□Quite often □Sometimes □Rarely □Never

6-17. Coloring or perming hair at a beauty salon after getting pregnant (check one only)

□Quite often □Sometimes □Rarely □Never

6-18. Use of sunscreen after getting pregnant (check one only)

□Quite often □Sometimes □Rarely □Never

6-19. Using drug for treatment of scabies or lice

□Yes □No
